# Budd-Chiari Syndrome: Long term success via hepatic decompression using transjugular intrahepatic porto-systemic shunt

**DOI:** 10.1186/1471-230X-10-25

**Published:** 2010-03-01

**Authors:** Alexandra Zahn, Daniel Gotthardt, Karl Heinz Weiss, Götz Richter, Jan Schmidt, Wolfgang Stremmel, Peter Sauer

**Affiliations:** 1 Department of Gastroenterology, University Hospital Heidelberg, Im Neuenheimer Feld 410, 69120 Heidelberg, Germany; 2 Department of Diagnostic Radiology, University Hospital Heidelberg, Im Neuenheimer Feld 110, 69120 Heidelberg, Germany; 3 Department of Surgery, University Hospital Heidelberg, Im Neuenheimer Feld 110, 69120 Heidelberg, Germany

## Abstract

**Background:**

Budd-Chiari syndrome (BCS) generally implies thrombosis of the hepatic veins and/or the intrahepatic or suprahepatic inferior vena cava. Treatment depends on the underlying cause, the anatomic location, the extent of the thrombotic process and the functional capacity of the liver. It can be divided into medical treatment including anticoagulation and thrombolysis, radiological procedures such as angioplasty and transjugular intrahepatic porto-systemic shunt (TIPS) and surgical interventions including orthotopic liver transplantation (OLT). Controlled trials or reports on larger cohorts are limited due to rare disease frequency. The aim of this study was to report our single centre long term results of patients with BCS receiving one of three treatment options i.e. medication only, TIPS or OLT on an individually based decision of our local expert group.

**Methods:**

20 patients with acute, subacute or chronic BCS were treated between 1988 and 2008. Clinical records were analysed with respect to underlying disease, therapeutic interventions, complications and overall outcome.

**Results:**

16 women and 4 men with a mean age of 34 ± 12 years (range: 14-60 years) at time of diagnosis were included. Myeloproliferative disorders or a plasmatic coagulopathy were identified as underlying disease in 13 patients, in the other patients the cause of BCS remained unclear. 12 patients presented with an acute BCS, 8 with a subacute or chronic disease. 13 patients underwent TIPS, 4 patients OLT as initial therapy, 2 patients required only symptomatic therapy, and one patient died from liver failure before any specific treatment could be initiated. Eleven of 13 TIPS patients required 2.5 ± 2.4 revisions (range: 0-8). One patient died from his underlying hematologic disease. The residual 12 patients still have stable liver function not requiring OLT. All 4 patients who underwent OLT as initial treatment, required re-OLT due to thrombembolic complications of the graft. Survival in the TIPS group was 92.3% and in the OLT group 75% during a median follow-up of 4 and 11.5 years, respectively.

**Conclusion:**

Our results confirm the role of TIPS in the management of patients with acute, subacute and chronic BCS. The limited number of patients with OLT does not allow to draw a meaningful conclusion. However, the underlying disease may generate major complications, a reason why OLT should be limited to patients who cannot be managed by TIPS.

## Background

Budd-Chiari syndrome (BCS) is a rare disorder defined as a hepatic venous outflow obstruction at any level between the hepatic veins and the right atrium [1] but generally implies thrombosis of the hepatic veins and/or the intrahepatic or suprahepatic inferior vena cava (IVC). Up to 50% of all cases of BCS are due to chronic myeloproliferative disorders like polycythemia vera (PV) [2] or coagulopathies like factor V (Leiden) gene mutation [3-5]. The clinical presentation is highly variable but may be categorized as acute and perhaps fulminant hepatic failure, as subacute without evidence of cirrhosis or as chronic with evidence of portal hypertension and cirrhosis.

Treatment depends on the underlying cause, the anatomic location, the extent of the thrombotic process and the severity of liver disease. Treatment options can be divided into medical treatment including anticoagulation and thrombolysis [6-8], radiological procedures such as angioplasty [9] and transjugular intrahepatic porto-systemic shunt (TIPS) [10-14] and surgical procedures including porto-systemic shunting (PSS) [15-17] and orthotopic liver transplantation (OLT) [18,19]. Anticoagulation alone is unlikely to lead to sufficient recanalization of occluded vessels, or development of adequate collateral circulation. However, satisfactory long-term survival with only medical therapy has been reported [6,20]. Recent data failed to show a favorable impact of PSS on survival [17,21], while TIPS has shown encouraging results [11-13,22-25].

This may give rise to redefine the role of OLT which may now be preserved for patients failing TIPS. The present treatment recommendations of BCS [26,27] are not based on randomized studies but on a small number of retrospective studies [8,25,27] and one prospective study [20].

The aim of this single centre retrospective study was to further enlarge the body of patients evaluated with the intention to allow steady optimization of present treatment strategies.

## Methods

### Study design

Patients admitted between 1988 and 2008 with a primary diagnosis of BCS were enrolled into this retrospective analysis. All available medical records especially laboratory data, radiological imaging and procedures, surgical interventions and discharge letters were reviewed. All data at the time of diagnosis and of new treatment were considered. Date of diagnosis was the date of the first investigation when the criteria for diagnosis were fulfilled. BCS was defined following the European network for vascular disorders of the liver (En-Vie) [28] criteria and the last Baveno consensus based on imaging showing an obstructed venous outflow tract [26]. Diagnosis of BCS was made by either Doppler ultrasonography, magnetic resonance imaging or computerized tomography.

Disease severity was defined as acute, subacute or chronic. In contrast to the acute disease, the subacute and chronic forms were assumed to be present for several weeks to more than six months prior to clinical presentation [29].

Different from the last Baveno consensus [26] treatment was only partly applied in a stepwise manner. On the basis of radiological imaging and the severity of clinical presentation a decision by an experienced interdisciplinary team concerning adequate treatment was reached. This could either be a medical treatment or a prompt intervention. If patients did not improve on medical therapy TIPS insertion was performed.

Patients who underwent OLT as initial therapy were not candidates for TIPS. Two patients were transplanted before TIPS had been introduced as a treatment option of BCS, namely in 1988 and 1992. The other patients had cirrhosis with signs of chronic liver failure in terms of hepatic encephalopathy and high bilirubin levels so that liver transplantation was considered essential in these patients.

### Hematological evaluation of hypercoagulable state

To identify a hypercoagulable state as the underlying etiology of BCS each patient received a comprehensive hematological evaluation. The latter was performed stepwise and included antiphospholipid antibodies, homocysteine levels, testing for factor V (Leiden) and prothrombin (20210) mutation, APC resistance, ATIII, protein C and S levels. Patients in whom a myeloproliferative disorder (MPD) like PV or essential thrombocytosis was suspected received a bone marrow biopsy. As our study begun in 1988, not all of our patients were screened for the JAK2V617F mutation.

### TIPS Technique and OLT

All TIPS were created using standard techniques [30-32] by insertion of Palmaz stents (Johnson and Johnson Interventional Systems, Warren, New Jersey), Wallstents (Schneider, Minneapolis, Minnesota) or covered Viatorr stents (GORE, Flagstaff, AZ). When a hepatic vein remnant was not present the portal vein was punctured directly from the IVC [30]. After the TIPS procedure patients underwent anticoagulation according to the guidelines [26]. Patients underwent control angiography 3 months after TIPS and in addition if shunt dysfunction was suspected. An abdominal ultrasound was performed every 6 months. TIPS dysfunction was defined as an increase in portosystemic gradient above 10 to 12 mmHg and clinical signs of portal hypertension.

In our liver transplantation program the modified piggyback technique by Belghiti [33] has been used as a routine surgical procedure since 2001. Before 2001, the so-called piggyback technique had been applied [34,35].

### Statistical analysis

Descriptive statistics were provided as mean ± standard deviation (SD) and as range. The cumulative survival probability was estimated by the method described by Kaplan and Meier. All analyses were carried out in Microsoft Excel and SPSS.

The study protocol conformed to the ethical guidelines of the Helsinki Declaration, and was approved by the ethics committee of the University of Heidelberg.

## Results

### Patients characteristics

20 patients, 4 male and 16 female, were included in our study. Patient age ranged from 14 to 60 with a mean age of 34 ± 12 years. Patients characteristics are given in table 1. Myeloproliferative disorders (especially PV) were the cause of BCS in 6 patients, 7 patients had plasmatic coagulation abnormalities, 1 patient took oestrogen medication as a possible underlying hypercoagulable condition and in 6 patients the aetiology of BCS remained unclear. As not all of our patients were screened for the JAK2V617F mutation latent MPD may have been missed in several patients. 12 patients presented with acute BCS, 8 with subacute or chronic disease. 8 patients presented with abdominal pain, 3 with new onset of ascites, 7 with abdominal pain plus new onset of ascites, 1 with gastrointestinal bleeding plus ascites and 1 with acute liver failure.

**Table 1:** Baseline Characteristics of all Patients

Patient	Underlying diagnosis	Clinical presentation	Time of presentation (year)	Time between primary diagnosis and intervention (months)	Relevant Comorbidities
**TIPS Group**					

1	Unknown	Abdominal pain	2006	0	None
2	Polycythemia vera	Ascites	2004	0	Thyroidectomy, Schizophrenia
3	Factor V (Leiden) mutation, protein C deficiency	Abdominal pain and ascites	2005	0	None
4	Unknown	Abdominal pain	1995	0	Myocardial infarction
5	Prothrombin mutation (20210)	Ascites	2002	0	Myasthenia gravis pseudoparalytica, Basedow disease
6	Polycythemia vera	Abdominal pain and ascites	1998	1	Arterial hypertension
7	Polycythemia vera	Abdominal pain and ascites	1996	2	Arterial hypertension, Atrial fibrillation
8	Antiphospholipid antibody syndrome, APC resistance	Abdominal pain	1995	1	Deep venous thrombosis
9	Unknown	Gastrointestinal bleeding and ascites	2001	1	None
10	Protein C and AT III deficiency	Abdominal pain	2005	0	None
11	APC resistance and AT III deficiency	Abdominal pain and ascites	2004	0	Atrial septal defect
12	Unknown	Abdominal pain	1993	48	None
13	Oestrogen medication	Abdominal pain and ascites	2007	0	None

**OLT Group**					

1	Polycythemia vera	Abdominal pain	1988	12	None
2	Protein C and S deficiency	Abdominal pain	2001	48	None
3	Unknown	Ascites	2003	36	Sarcoidosis
4	Unknown	Abdominal pain and ascites	1992	24	Osteoporosis

**Non-intervention Group**					

1	Myeloproliferative Disorder	Acute liver failure	2005		None
2	Factor V (Leiden) mutation	Abdominal pain and ascites	2003		None
3	Essential thrombocythemia	Abdominal pain	2003		None

### Treatment and outcome

13 patients underwent TIPS (10 women, 3 men) and 4 underwent OLT (3 women, 1 man) as initial therapy. One of the remaining 3 patients died of fulminant liver failure rapidly after the initial diagnosis, the other patients have not had an intervention, yet as their status was stable under a symptomatic anticoagulation therapy. In the TIPS group mean age was 36 ± 13 years (range: 20-60 years) and in the transplant group 27 ± 9 years (range: 14-34 years). 8 of 13 patients in the TIPS group underwent TIPS within one week after the diagnosis of BCS was confirmed, 3 patients underwent TIPS within 1 month, 1 patient within 2 months and in 1 patient time period until TIPS placement was 48 months.

The porto-systemic pressure gradient was lowered by a mean of 21 ± 10 mmHg (range: 6-40 mmHg) in the 13 patients initially treated with TIPS. After a median follow-up of 4 years (range: 6 months to 12 years) 11 of these 13 patients (85%) developed TIPS dysfunction requiring reintervention. In most cases TIPS dysfunction was due to thrombosis or pseudointimal hyperplasia and could be managed by dilation. On average, 2.5 ± 2.2 revisions per patient were necessary (range: 0-8) (see table 2). 12 out of 13 patients survived within the follow up period (92%). One patient died in the course of the underlying hematologic disease. After TIPS placement no patient developed clinical relevant hepatic encephalopathy. None of the patients in the TIPS group had to undergo OLT subsequently.

**Table 2:** Angiographic findings and interventions in the TIPS group

Patient	Angiographic findings*	gradient reduction**	Number of additional Revisions	Follow up
1	HV, PV, SMV occluded	17 → 8	2	2 years
2	HV occluded	30 → 7	1	4 years
3	HV occluded	23 → 10	3	2 years
4	HV occluded	29 → 12	2	12 years
5	HV occluded	46 → 6	1	5 years
6	HV, PV, IVC occluded	35 → 6	8	7 years
7	HV occluded	32 → 6	4	11 years
8	HV occluded	25 → 10	1	3 years
9	HV occluded	20 → 8	2	6 years
10	HV occluded	32 → 3	0	3 years
11	HV occluded	31 → 9	3	3 years
12	HV occluded	10 → 4	6	10 years
13	HV occluded	38 → 6	0	1/2 year

*HV: hepatic veins; PV: portal vein; SMV: superior mesenteric vein; IVC: inferior vena cava

**Numbers indicate original portosystemic pressure gradient followed by post-TIPS gradient in mmHg.

After a mean of 30 ± 13 months after diagnosis (range: 12-48 months) patients in the transplant group underwent OLT. Each patient who underwent OLT as initial therapy, had to undergo re-OLT. The first patient had retransplantation within a few days because of portal vein thrombosis and graft failure. Today, 20 years after re-OLT she is still alive and has an excellent graft function. The second patient had to undergo 2 retransplantations. She had ischemic cholangiopathy in all explants and in the second explant ischemic cholangiopathy in relation with arterial thrombosis was found, histologically. The patient died due to septic complications 3 months after the third OLT. The third patient also had retransplantation within a few days because of vascular obliteration of the hepatic artery and a necrosis of the common bile duct. This patient is still alive without further complications. The fourth patient had to undergo retransplantation 9 months after initial OLT because of ischemic graft failure. She is still alive 15 years after OLT without limitations. Although no recurrence of BCS in the transplants has been detected, each patient in the transplant group suffered from various thrombotic complications. Within the observation period 3 out of 4 patients survived (75%).

**Figure 1:**
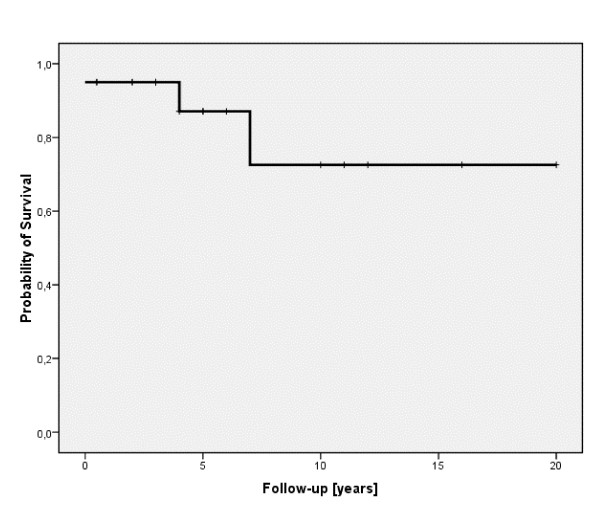
**Probability of survival given for all BCS patients**. On the x-axis the time of follow-up in years is given. On the y-axis the probability of survival is shown.

The overall probability of survival on an intention to treat basis for the whole cohort of 20 patients with BCS was 72.6% after a median follow-up of 5 years (range: 0 to 20 years), (see figure 1).

## Discussion

In this study, we assessed the clinical course and long term outcome of patients with BCS referred to our university hospital analysing the data of all patients admitted with a primary diagnosis of BCS between 1988 and 2008.

Sequential use of increasingly invasive procedures based upon the clinical response has been recommended for the treatment of BCS [8,25,27]. In one study in 14 patients, treated with diuretics and anticoagulation alone, a mortality rate of 86% during a period of 6 months was reported [36], however acceptable long term survival rates with only medical therapy has been reported in other trials [37]. Surprisingly, in the recently published first prospective study [20] nearly 50% of the patients were managed conservatively, too. But there may be limitations of this study; on the one hand the study was not restricted to patients with severe disease and on the other hand the median follow-up was only 17 months. In our present trial only 2 patients have not had an invasive therapeutic procedure and are still without clinical symptoms under anticoagulation therapy. Both patients have incomplete hepatic venous outflow obstruction and presented with chronic or subacute BCS. In addition, in one of these two patients the follow-up period of half a year is relatively short. On the basis of this very limited experience, we may confirm the value of medical treatment alone in patients with limited disease. In contrast, in the patients with severe disease or symptomatic cirrhosis, medical treatment was not effective and interventional treatment (TIPS) or transplantation had to be applied.

Different from the last Baveno consensus [26] treatment was only partly applied in a stepwise manner. On the basis of radiological imaging and the severity of clinical presentation a decision concerning adequate treatment was reached. In our collective 13 patients underwent TIPS as initial therapy. In the follow-up, one patient died in the course of the underlying hematologic disease so that an overall survival of 92% was achieved. In all patients transplantation could be avoided. Similar 5 year transplant free survival rates between 77 and 100% after TIPS were observed in other studies [11-13,22-25]. These results seem to justify primary TIPS treatment in patients with severe disease without awaiting failure of a previous treatment step. Both the high survival and the prevention of OLT in the patients initially treated with TIPS demonstrate an advantage over a stepwise strategy recommended by Baveno and applied in the study by Plessier et al. [8]. Although, the latter found high survival rates using therapeutic procedures by order of increasing invasiveness, the need for transplantation after TIPS was high (38%) compared to our study. This may be due to the fact that TIPS is more efficient when inserted early. Therefore, our results suggest that TIPS should no longer be considered as a bridge to OLT but as a definitive treatment option in BCS, especially when prompt intervention and a high grade of interventional experience is available. With regard to the current situation of liver donor shortage these results are important.

Although, our protocol involved resumption of anticoagulation immediately after TIPS placement, 85% of our patients had at least one reintervention within a mean follow-up of 6 years. In all these patients TIPS revision was technically successful and effective. However, TIPS dysfunction seems to be a common problem [38,39]. Since, covered stents have a considerable advantage over bare stents with a lower dysfunction rate for the treatment of BCS patients [40], covered stents should be preferred.

Four patients underwent OLT as initial therapy. Two were transplanted before TIPS had been introduced as a treatment option of BCS and two had cirrhosis with signs of chronic liver failure. Survival following OLT depends upon the underlying cause of BCS and the patients condition at the time of transplantation [41]. A large series with 510 patients [42] found a 3 year patient survival of 85% after introduction of the Model for End-Stage Liver Disease (MELD) score versus a 3 year patient survival of 73% in the pre-MELD era. In two other studies [41,43] 10 year survival rates were reported between 69 and 68%, respectively. It has been suggested, that the outcome of OLT in BCS patients does not differ from that of other etiologies of liver failure when adequate longterm anticoagulation is administered [44]. Even in patients with myeloproliferative diseases survival rates are similar to those in patients with other underlying etiologies [41]. Although, the number of patients transplanted in the present trial does not allow any conclusions on survival, the overall survival rate of 75% during a mean follow-up period of 11 years is comparable to the reported results.

Each patient who underwent OLT as initial therapy, had to undergo re-OLT, which is one of the most important results of our study. All patients had graft failure due to thrombotic or vascular complications. These complications have been described in other series and severe thrombotic complications occurred despite routine early posttransplantation anticoagulation [8,41]. In accordance to other reports, these findings support the concept that aggressive anticoagulation as early as possible may reduce the risk of thrombembolic complications as well as the late recurrence of BCS after OLT [41,43].

However, it seems likely, that in some cases thrombembolic complications will not be preventable, despite of aggressive anticoagulation as observed in one patient in our series.

## Conclusion

In conclusion, therapy for BCS usually requires TIPS or OLT. The treatment modality is dependent on duration of illness, extent of thrombosis and degree of liver dysfunction. TIPS is successful as initial therapy as it promotes clinical improvement in the long run even though shunt revisions are generally needed. In the present study the necessity of subsequent OLT was remarkably low and thus TIPS could be regarded as definitive treatment option in BCS. OLT in BCS is associated with an increased risk of thrombembolic complications and early graft failure in spite of consequent anticoagulation therapy.

## Competing interests

The authors declare that they have no competing interests.

## Authors' contributions

AZ contributed substantially to the design of the study, performed data collection, data analysis and wrote the paper. DG contributed to the data collection and analysis. KHW contributed to the data collection and analysis. GR performed the radiological interventions, contributed to the data collection and analysis. JS contributed to the data collection and analysis concerning the transplant patients. WS contributed substantially to the design of the study and to the interpretation of data. PS developed the original idea of the study, was involved in data analysis and reviewed the manuscript finally. All authors read and approved the final manuscript.

## Pre-publication history

The pre-publication history for this paper can be accessed here:

http://www.biomedcentral.com/1471-230X/10/25/prepub
